# Assembling Polyiodides and Iodobismuthates Using a Template Effect of a Cyclic Diammonium Cation and Formation of a Low-Gap Hybrid Iodobismuthate with High Thermal Stability

**DOI:** 10.3390/molecules25122765

**Published:** 2020-06-15

**Authors:** Tatiana A. Shestimerova, Andrei V. Mironov, Mikhail A. Bykov, Anastasia V. Grigorieva, Zheng Wei, Evgeny V. Dikarev, Andrei V. Shevelkov

**Affiliations:** 1 Department of Chemistry, Lomonosov Moscow State University, 119991 Moscow, Russia; shestimerova@inorg.chem.msu.ru (T.A.S.); avmironov@inorg348-1.chem.msu.ru (A.V.M.); mich.bykov@gmail.com (M.A.B.); anastasia.grigorieva@gmail.com (A.V.G.); 2 Department of Materials Sciences, Lomonosov Moscow State University, 119991 Moscow, Russia; 3 Department of Chemistry, University at Albany, Albany, NY 12222, USA; zwei@albany.edu (Z.W.); edikarev@albany.edu (E.V.D.)

**Keywords:** template effect, iodometallates, bismuth, polyiodides, crystal structure, intermolecular interactions, optical properties

## Abstract

Exploiting a template effect of 1,4-diazacycloheptane (also known as homopiperazine, Hpipe), four new hybrid iodides, (HpipeH_2_)_2_Bi_2_I_10_·2H_2_O, (HpipeH_2_)I(I_3_), (HpipeH_2_)_3_I_6_·H_2_O, and (HpipeH_2_)_3_(H_3_O)I_7_, were prepared and their crystal structures were solved using single crystal X-ray diffraction data. All four solid-state crystal structures feature the HpipeH_2_^2+^ cation alternating with Bi_2_I_10_^4–^, I_3_^–^, or I^–^ anions and solvent water or H_3_O^+^ cation. HpipeH_2_^2+^ assembles anionic and neutral building blocks into polymer structures by forming four strong (N)H···I and (N)H···O hydrogen bonds per cation, with the H···I distances ranging from 2.44 to 2.93 Å and H···O distances of 1.88–1.89 Å. These hydrogen bonds strongly affect the properties of compounds; in particular, in the case of (HpipeH_2_)_2_Bi_2_I_10_·2H_2_O, they ensure narrowing of the band gap down to 1.8 eV and provide high thermal stability up to 240 °C, remarkable for a hydrated molecular solid.

## 1. Introduction

Bismuth is the heaviest chemical element to possess stable isotopes [1]. For decades, it has amused chemists by an astonishing variety of its molecular and condensed clusters that have no analogues among lighter elements [2,3,4,5,6,7,8]. Recently, other compounds of bismuth have become the objects of rapt attention. Today, halobismuthates are scrutinized as potential solar light absorbers to replace their efficient but toxic lead congeners. Although the photovoltaic efficiency of Bi-based solar cells has so far reached only 2.1%, the search for new materials is gaining interest and new compounds have been recently synthesized and examined for potential photovoltaic properties [9,10,11,12,13,14,15]. In general, halobismuthates fulfill basic requirements for solar light harvesting. The Bi^3+^ cation possesses a polarizable electron shell prone to spin-orbit coupling, and its derivatives are stable against reduction and oxidation in addition to showing very low toxicity. However, in order to be good candidates, halobismuthates should also possess low band gaps; therefore, band gap engineering comes to the fore in creating Bi-based materials for solar light harvesting.

Bismuth(III) forms quite a number of iodide complexes with various cations [16,17]. Inorganic compounds of a general formula A_3_Bi_2_I_9_, where A is a univalent cation, crystallize into two basic structure types. One of those features is a layered structure based on corner-shared [BiI_6_] octahedra, whereas [Bi_2_I_9_]^3–^ bioctahedra are the building blocks in another type. Both types were reported to have band gaps in the vicinity of 2 eV as well as low photovoltaic efficiency. Other compounds with inorganic cations contain solvent water. Their band gaps are narrower, approaching 1.75 eV, and almost independent of the nature of the inorganic cation [18,19,20]. The major obstacle in the way of their application is low thermal stability; these compounds are known to decompose around 100 °C. The band gap of iodobismuthates can be significantly reduced when bismuth iodide is combined with iodides of copper or silver. Several heterometallic iodides of this kind with different crystal structures have been prepared to date and their photovoltaic properties have been tested. It was shown that the gap width covers a wide range (1.5–2.5 eV), whereas the light-to-current efficiency depends on the structure and composition and varies from 0.83 to 3.17% [21,22,23,24,25,26,27]. Another route to low band gap iodobismuthates is introduction of I_2_ or I_3_^–^ moieties into the crystal structures. In this way, several compounds with the band gap width around 1.5 eV were recently synthesized [28,29,30,31]; however, their low thermal stability may severely limit potential applications.

Hybrid organic–inorganic iodobismuthates, as a rule, display moderate thermal stability, with decomposition temperatures near 200 °C, even if a solvent is not incorporated into their structure. At the same time, the majority of these compounds have rather wide band gaps exceeding 2 eV, which makes them poor light harvesters. However, recent literature provides several examples of hybrid iodobismuthates with low band gaps, ranging from 1.59 to 1.80 eV [32,33,34,35,36]. These compounds contain various organic cations and, more importantly, have different dimensionality of the anionic substructure, ranging from isolated anions to vertex-sharing BiI_5_ chains to edge-sharing BiI_4_^–^ chains. The analysis of the crystal structures of hybrid iodobismuthates shows that features other than the dimensionality of the Bi/I substructure play an important role in controlling the band gap width. Namely, they are multifold weak interactions that include hydrogen bonds (mainly (N)H···I) as well as interanionic I···I interactions [36,37,38,39,40,41,42]. DFT calculations reveal that weak non-covalent bonds are capable of promoting charge mobility along the direction of spreading such bonds in the crystal structure [34,35,36,37,38,39].

We propose that a strong organic base capable of forming hydrogen bonds with the [BiI_6_] octahedra will favor the formation of a low band gap hybrid iodobismuthate irrespective of the dimensionality of the anionic Bi/I substructure. In this work, we exploit the template effect of 1,4-diazacycloheptane (also known as homopiperazine, Hpipe) to synthesize a hybrid iodobismuthate (HpipeH_2_)_2_Bi_2_I_10_·2H_2_O with a band gap of 1.8 eV. We describe its crystal structure as well as of three other new compounds, (HpipeH_2_)_3_I_6_·H_2_O, (HpipeH_2_)I(I_3_), and (HpipeH_2_)_3_(H_3_O)I_7_, that also manifest the same template mode of Hpipe. We were particularly concerned with the pattern of hydrogen bonds in all four compounds as well as the high thermal stability and promising optical properties of (HpipeH_2_)_2_Bi_2_I_10_·2H_2_O.

## 2. Results

(HpipeH_2_)_2_Bi_2_I_10_·2H_2_O was synthesized in the form of a dark-red polycrystalline powder by a reaction between diluted hydroiodic acid solutions of 1,4-diazacycloheptane and BiI_3_. Upon washing with water and drying in air, the precipitate was analyzed for phase purity by means of powder X-ray diffraction, which showed a perfect match of the observed diffraction pattern with that calculated from the single crystal data (see Figure S1 of Supplementary Materials). The compound is stable for at least several weeks at ambient conditions. According to the thermal analysis (see Figure S2 of Supplementary Materials), its thermal decomposition starts upon heating to 240(2) °C releasing water, organic fragments, and then BiI_3_, which is known to be volatile above 300 °C.

In the absence of bismuth, Hpipe itself reacts with hydroiodic acid yielding different products depending on the concentration of the acid and on the presence of additional iodine in the solution. Yellowish-white (HpipeH_2_)_3_I_6_·H_2_O is readily obtained upon reacting Hpipe with 14% HI. With higher concentration of the acid (27%), the reaction involves partial oxidation of HI by air oxygen, ultimately resulting in the formation of brown (HpipeH_2_)I(I_3_). When an excess of 14% HI is used, (HpipeH_2_)_3_I_6_·H_2_O transforms into (HpipeH_2_)_3_(H_3_O)I_7_, which can be isolated as a yellow polycrystalline powder. Its reaction with 27% HI involves partial oxidation of hydroiodic acid, yielding (HpipeH_2_)I(I_3_).

The crystal structure of each of the compounds described was determined from a single crystal. The summary of experimental and crystallographic information for studied compounds is given in Table 1. Selected interatomic distances are given in Table 2, with hydrogen bonding in Table 3.

**Figure 1:**
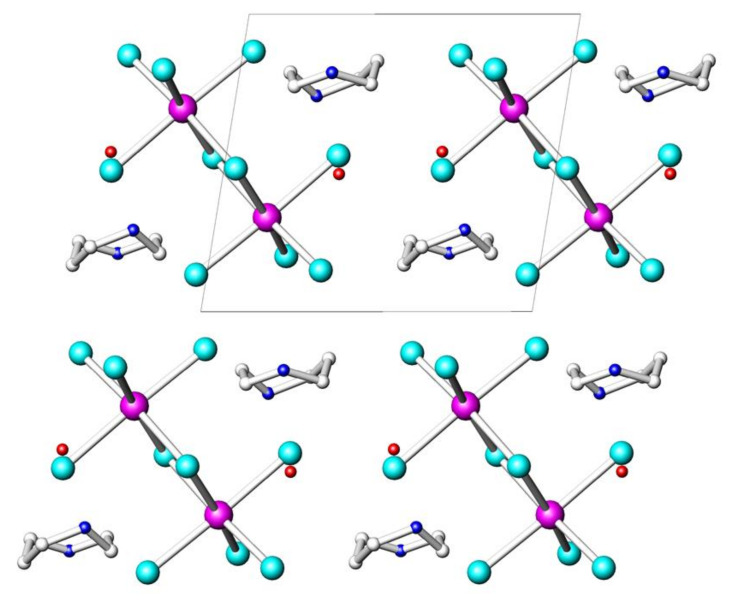
Projection of the crystal structure of (HpipeH_2_)_2_Bi_2_I_10_·2H_2_O onto (100) plane. Bismuth, magenta; iodine, cyan; oxygen, red; nitrogen, blue; carbon, light gray. Hydrogen atoms are omitted for clarity. Unit cell is shown by thin black lines.

The crystal structure of (HpipeH_2_)_2_Bi_2_I_10_·2H_2_O features three basic building blocks; they are the [Bi_2_I_10_]^4–^ anion, the HpipeH_2_^2+^ cation, and water as an interstitial solvent (Figure 1). The Bi_2_I_10_^4–^ anion is an edge-shared bioctahedron; such a structural unit is frequently observed in various iodobismuthates [16,17]. The Bi–I distances to the bridging iodine atoms, 3.1459(10) and 3.1946(17) Å, are slightly longer than the Bi–I bonds to terminal iodine atoms, 3.0344(9) and 3.1227(10) Å (Table 2). The anions are linked to the HpipeH_2_^2+^ cations via hydrogen (N)H···I bonds (Table 3). The (N)H···I interatomic distances of 2.72–2.77 Å are remarkably short compared to the hydrogen bond distances of 2.87 Å and greater than that observed for similar contacts in various compounds [39,43], whereas the (O)H···I distance of 2.79(9) Å is usual for such kind of hydrogen bonds. The N–H···I and O–H···I angles substantially deviate from linearity, as typically encountered in similar assemblies [43,44]. In addition, there are numerous (C)H···I interatomic distances in the crystal structure, with the H···I separation ranging from 3.13 to 3.41 Å; the former distance may point at a very weak hydrogen bond, whereas the latter one is even greater than the sum of the respective van-der-Waals radii of 3.24 Å [45]. Finally, the I···I separations between the iodine atoms of the neighboring [Bi_2_I_10_]^4–^ anions exceed 4.14 Å. These are considerably longer than the I···I distances of 3.7–3.9 Å, which were reported to have an impact on the electronic structure of iodobismuthates [28,36,39] and are greater than the doubled van-der-Waals radii of iodine (4.08 Å [45]).

**Figure 2:**
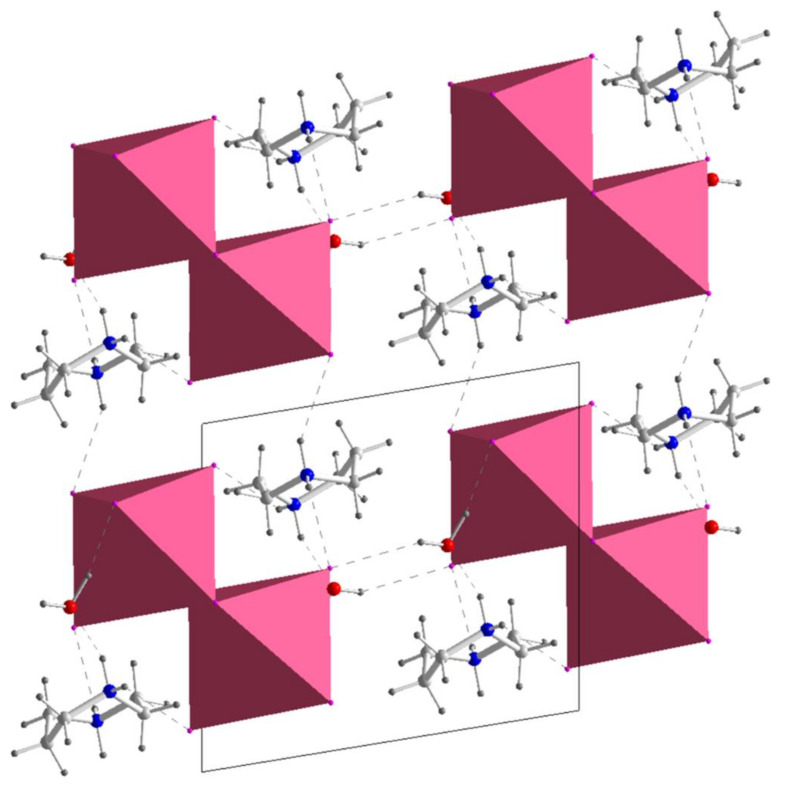
View of the crystal structure of (HpipeH_2_)_2_Bi_2_I_10_·2H_2_O along the *c* axis. Bi_2_I_10_^4–^ octahedra, magenta; oxygen, red; nitrogen, blue; carbon, light gray; hydrogen, dark gray. Hydrogen (N)H···O, (O)H···I, and (N)H···I bonds are shown as dashed lines; the unit cell is depicted by thin black lines.

The HpipeH_2_^2+^ dication serves as a template to organize Bi_2_I_10_^4–^ anions and solvent water into a 3D crystal network. Each nitrogen atom of HpipeH_2_^2+^ has two hydrogen atoms and employs them to form hydrogen bonds with the anionic part. In the crystal structure of (HpipeH_2_)_2_Bi_2_I_10_·2H_2_O, one nitrogen atom is involved in two (N)H···I bonds with iodine atoms of two different Bi_2_I_10_^4–^ anions, whereas another nitrogen atom forms one hydrogen bond of the same type and a (N)H···O hydrogen bond of 1.88 Å with solvent water, which is quite short compared to other organoammonium derivatives [40]. In this way, the HpipeH_2_^2+^ dication becomes surrounded by three Bi_2_I_10_^4–^ anions and one water molecule. The alternation of such hydrogen bonds throughout the crystal structure leads to the 3D pattern shown in Figure 2.

**Figure 3:**
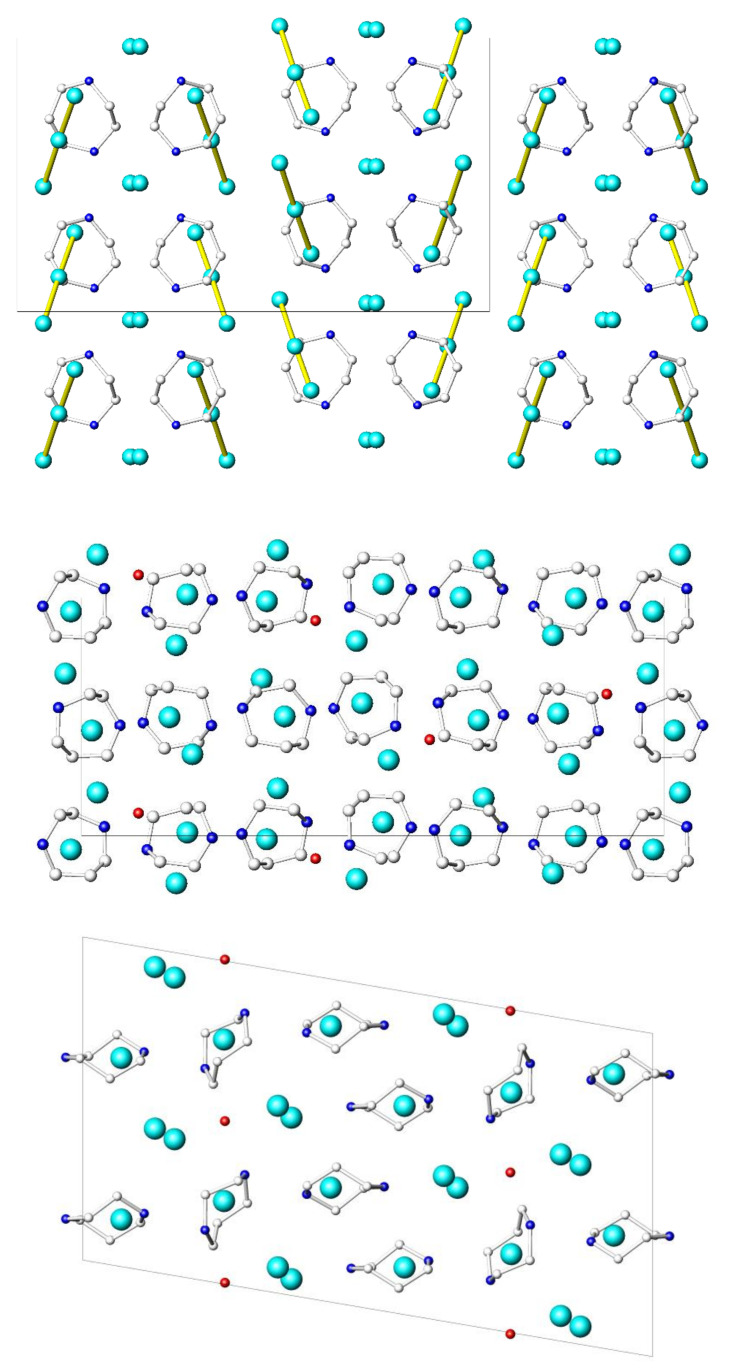
Projections of the crystal structures of (HpipeH_2_)I(I_3_) on the [010] plane (top), (HpipeH_2_)_3_I_6_·H_2_O on the [100] plane (middle), and (HpipeH_2_)_3_(H_3_O)I_7_ on the [010] plane (bottom). Iodine, cyan; oxygen, red; nitrogen, blue; carbon, light gray. Hydrogen atoms are omitted for clarity. Black lines show unit cells.

**Figure 4:**
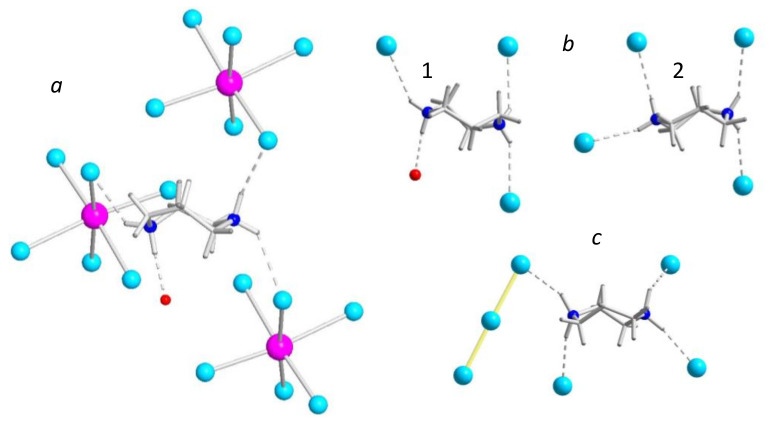
The surroundings of HpipeH_2_^2+^ cations in the crystal structures of (HpipeH_2_)_2_Bi_2_I_10_·2H_2_O (**a**), (HpipeH_2_)_3_I_6_·H_2_O (**b**), and (HpipeH_2_)I(I_3_) (**c**). Bismuth, magenta; iodine, cyan; oxygen, red; nitrogen, blue; carbon, light gray, hydrogen, dark gray. Hydrogen (N)H···O and (N)H···I bonds are shown as dashed lines. Note that the surrounding of HpipeH_2_^2+^ cations in the crystal structure of (HpipeH_2_)_3_(H_3_O)I_7_ is the same as in (HpipeH_2_)_3_I_6_·H_2_O, type 2 (b).

**Figure 5:**
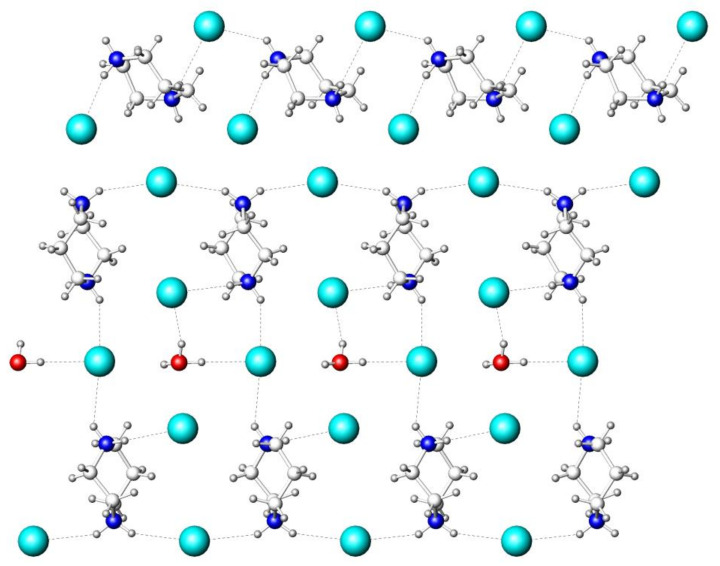
Pattern of hydrogen bonds in the crystal structure of (HpipeH_2_)_3_(H_3_O)I_7_ involving HpipeH_2_^2+^ and H_3_O^+^ cations and iodide anions. Two strands running along the *c* axis are shown. Iodine, cyan; oxygen, red; nitrogen, blue; carbon, light gray, hydrogen, dark gray. Hydrogen (N)H···O and (N)H···I bonds are shown as dashed lines.

The propensity of HpipeH_2_^2+^ to form four hydrogen bonds manifests itself in three other crystal structures that are revealed in this work (Figure 3). In homopiperazinium iodide-triiodide, (HpipeH_2_)I(I_3_), the HpipeH_2_^2+^ cation forms four (N)H···I bonds, of which three connect the dication with monoatomic I^–^ anions, whereas the forth bond links the dication with the triiodide anion I_3_^–^ through the terminal iodine atom (Figure 4). The crystal structure of homopiperazinium iodide monohydrate, (HpipeH_2_)_3_I_6_·H_2_O, is a bit more complex. It shows two modes of the HpipeH_2_^2+^ cation involvement in hydrogen bonding. The first one is essentially the same as in (HpipeH_2_)_2_Bi_2_I_10_·2H_2_O, where three iodine atoms and one water molecule assemble around HpipeH_2_^2+^. The second mode is coordination of four I^–^ anions by (N)H···I bonds. Finally, the HpipeH_2_^2+^ cation forms four (N)H···I bonds in the crystal structure of (HpipeH_2_)_3_(H_3_O)I_7_; however, the latter crystal structure features a more complex array of hydrogen bonds as it involves the H_3_O^+^ oxonium cation that forms (O)H···I bonds with three I^–^ anions, leading to the 1D pattern of hydrogen bonds shown in Figure 5. The H_3_O^+^ cation is involved only in (O)H···I bonds, which makes its role different from that in the crystal structure of (H_3_O)Rb_3_BiI_7_·4H_2_O. In the latter, the H_3_O^+^ cation, along with water molecules, forms a 1D (H_11_O_5_)^+^ polymeric cation, which takes part in interactions with Rb^+^ cations [46].

**Figure 6:**
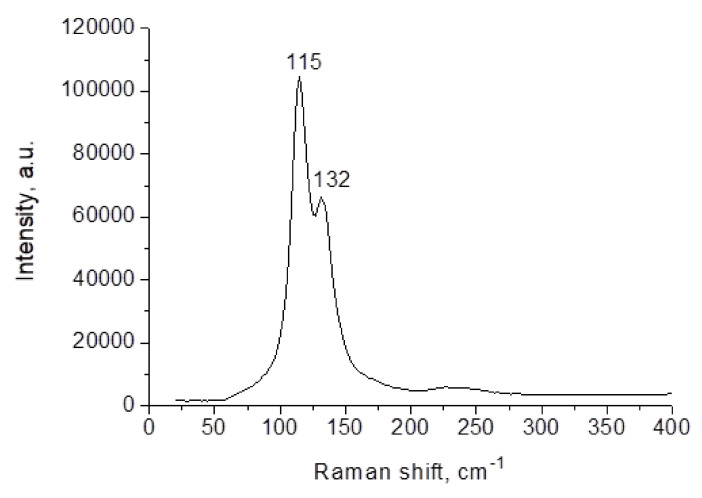
Raman spectrum of (HpipeH_2_)I(I_3_).

The length of the (N)H···I bonds in (HpipeH_2_)I(I_3_), (HpipeH_2_)_3_I_6_·H_2_O, and (HpipeH_2_)_3_(H_3_O)I_7_ covers the range of 2.44–2.93 Å, which is considerably wider than the range of the respective distances found in (HpipeH_2_)_2_Bi_2_I_10_·2H_2_O (2.72–2.77 Å). The distance of 2.44(10) Å is remarkably short for the (N)H···I bond. Strong hydrogen bonds influence the geometry of the I_3_^–^ anion in the crystal structure of (HpipeH_2_)I(I_3_). This anion is asymmetric; the I–I distances are 2.8679(8) and 2.9651(8) Å and the I–I–I angle is 178.82(2) deg. Such asymmetry stems from the binding mode of the I_3_^–^ anion, where only one terminal atom forms a short (N)H···I bond of 2.56(19) Å, and is manifested in the Raman spectrum (Figure 6), which features two bands that can be assigned to symmetric and asymmetric stretching modes [47]. The former is observed at 115 cm^–1^ and is supplemented by scarcely visible overtone satellites at about 230 and 340 cm^–1^. It has a slightly higher Raman shift than normally observed for the triiodide anion (110 cm^–1^), [48,49] but that can be explained by a slightly shorter average I–I interatomic distance (2.92 Å) compared to what is typical for the I_3_^–^ anion (2.94 Å) [50]. The band at 132 cm^–1^ should be attributed to the asymmetric stretch mode, which is typically observed in the range of 130–140 cm^–1^. For the I_3_^–^ anion adopting *D_∞h_* symmetry, this vibration is Raman-inactive; however, the intensity of this band grows with the degree of the I_3_^–^ anion deviation from the *D_∞h_* symmetry.

We would like to emphasize that most of the hydrogen bonds in the four title crystal structures are quite short. The lengths apparently reflect the strength of such bonds, most likely associated with the base strength of Hpipe. A considerable number of strong hydrogen bonds ensures assembling of the molecular units into patterns infinite in one, (HpipeH_2_)_3_(H_3_O)I_7_, two, (HpipeH_2_)_3_I_6_·H_2_O and (HpipeH_2_)I(I_3_), or three, (HpipeH_2_)_2_Bi_2_I_10_·2H_2_O, directions. The latter compound exhibits rather high thermal stability up to 240 °C, quite remarkable for a hydrated molecular solid. One can argue that the main reason for such stability is a 3D pattern of rather strong hydrogen bonds (Figure 2).

**Figure 7:**
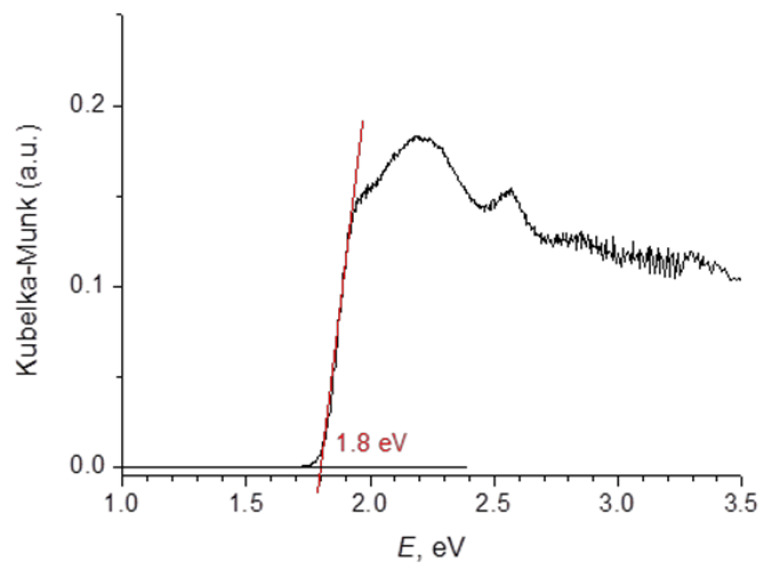
Kubelka–Munk plot for (HpipeH_2_)_2_Bi_2_I_10_·2H_2_O.

Optical diffuse reflectance measured for a polycrystalline powder of (HpipeH_2_)_2_Bi_2_I_10_·2H_2_O and converted into absorbance data is plotted versus energy in Figure 7. Extrapolation of the linear part of the Kubelka–Munk plot onto the energy axis gives the value of a direct band gap of 1.8 eV, which is in line with the dark-red color of the compound. It was previously shown that the charge transfer in iodobismuthates proceeds from iodine 5*p* orbitals lying at the top of the valence band to bismuth 6*p* orbitals forming the bottom of the conduction band [19]. However, the structure of the top of the valence band and the bottom of the conduction band may be affected by additional contributions. Two of those, namely, I···I secondary interactions and polyiodide moieties can be ruled out as absent in the crystal structure of (HpipeH_2_)_2_Bi_2_I_10_·2H_2_O. The remaining factor influencing the band gap width is the interaction between the cation and anion. Unlike the case of (C_7_H_7_)BiI_4_, in which the cation-to-anion charge transfer takes place [51], the shrinkage of the band gap in the title compound can be attributed to the contribution of hydrogen bonds. Firstly, strong (N)H···I hydrogen bonds slightly push iodine 5*p* orbitals to higher energy rendering smaller width of the ban gap; secondly, hydrogen bonds are capable of promoting mobility of charge carriers, ensuring 3D conductivity in solids composed of 0D Bi/I anions [34,39,52,53]. The stronger the non-covalent (hydrogen) bond is, the higher the mobility should be. In (HpipeH_2_)_2_Bi_2_I_10_·2H_2_O, very strong hydrogen bonds provoke reduction of the band gap from 2.0–2.2 eV, typical for compounds built of 0D Bi/I anions [53,54], to as low as 1.8 eV. The latter ensures efficient absorption of the solar light, which is favorable for the creation of light-harvesting materials for new solar cells.

## 3. Materials and Methods

### 3.1. Synthesis

Used as starting materials were Bi (granules, 99.99%), I_2_ (analytical grade), 1,4-diazacycloheptane (analytical grade), P (pure), and H_2_O (distilled). BiI_3_ was synthesized from the elements, and hydroiodic acid (stabilized) was synthesized by hydrolysis of freshly prepared PI_3_; details of these procedures are described elsewhere [19]. The HI acid (stabilized) was distilled at 126 °C, and the resulting solution was diluted with distilled water to required concentrations.

For the preparation of (HpipeH_2_)_2_Bi_2_I_10_·2H_2_O, 10 ml of aqueous solution containing 10 wt.% of HI was added to the starting reagents taken in a molar ratio of BiI_3_:Hpipe = 1:1 with a total weight of 0.2 g. The flask with the resulting solution was left for evaporation in open air for five days to yield dark-red well-shaped crystals. These crystals were separated by filtration and dried at room temperature. A mixture of large yellow crystals of (HpipeH_2_)_3_I_6_·H_2_O and large column-shaped brown crystals of (HpipeH_2_)I(I_3_) was obtained by slow evaporation of a homopiperazine solution in a 27 wt.% of HI. Pure compounds of these compositions were obtained by the following procedures: For the preparation of (HpipeH_2_)_3_I_6_·H_2_O, 4 ml of aqueous solution containing 14 wt.% of HI was added to 0.5 g of Hpipe. Yellowish crystals formed immediately. For the preparation of (HpipeH_2_)I(I_3_), 10 ml of the aqueous solution containing 50 wt.% of HI were added to a mixture of 1 g of Hpipe and 0.5 g I_2_ and the resulting solution was kept in air until complete evaporation of the solvent, which yields brown crystals of the target compound. (HpipeH_2_)_3_(H_3_O)I_7_ was obtained by adding a 10-fold excess of HI (14 wt.%) to the aqueous solution of Hpipe. The resulting solution was evaporated almost to dryness to yield yellowish-white (HpipeH_2_)_3_(H_3_O)I_7_ contaminated by a minor impurity of (HpipeH_2_)_3_I_6_·H_2_O.

### 3.2. Powder X-Ray Diffraction Analysis (PXRD)

Powder X-Ray diffraction analysis (PXRD) was performed on an Imaging Plate Guinier Camera (Huber G670, Cu-K*_α_*_1_ radiation, *λ* = 1.540598 Å, Huber Diffraktionstechnik GmbH & Co. KG, Rimsting, Germany) with the 2θ ranging from 5 to 77 deg. For the analysis, crystals were finely crushed in an agate mortar, and the resulting powder was fixed on a holder using scotch tape.

### 3.3. Crystal Structure Determination

Single crystals of (HpipeH_2_)_2_Bi_2_I_10_·2H_2_O were selected directly from the synthetic mixture. Single crystal diffraction data were measured on a CAD4 diffractometer equipped with an Ag Kα X-ray tube. The experimental data were corrected for Lorenz and polarization factors and absorption effect. The structure was solved by direct methods (*SIR2002* program package) [55]. The solution of the crystal structure revealed bismuth and iodine atoms arranged in pairs of edge-sharing [BiI_6_] octahedra. Structure refinement and successive Fourier synthesis (JANA 2000 [56]) revealed C_5_N_2_ rings and additional separate atoms, which were interpreted as water oxygens. Hydrogen atoms near carbons were placed geometrically and refined constrained in a riding mode. Difference Fourier synthesis in the vicinity of nitrogen atoms showed several residual peaks at distances corresponding to hydrogen atoms. Being refined independently they appeared in reasonable (but not perfect) positions with good atomic displacement parameters. For the final refinement, they were placed geometrically and refined constrained. The additional proof of such an approach is the charge neutrality of the structure. At the final stage, the oxygen atom was replaced by the water molecule, which was refined as a rigid body with fixed interatomic distances and angles.

Well-shaped single crystals of (HpipeH_2_)_3_I_6_·H_2_O and (HpipeH_2_)I(I_3_) were selected from the respective synthetic mixtures. The X-ray intensity data were measured at 293 K on a STOE StadiVari Pilatus 100 K diffractometer system equipped with a Cu-target X-ray tube (λ = 0.56083 Å) and a graphite monochromator (Stoe & Cie GmbH, Darmstadt, Germany). The crystals were mounted on a goniometer head with epoxy glue. The detector was placed at a distance of 80 mm from the crystal. A total of 1113 and 4142 frames were collected for (HpipeH_2_)_3_I_6_·H_2_O and (HpipeH_2_)I(I_3_), respectively. The frames were integrated, and absorption correction was performed with the STOE X-AREA Software package. The structure was solved and refined by full-matrix least-squares procedures on |*F^2^*| using the SHELX97 software [57]. The coordinates of iodide and several Hpipe atoms were found by the direct method. The remaining Hpipe atoms and oxygen atoms (in the case of (HpipeH_2_)_3_I_6_·H_2_O) were found in the successive difference Fourier syntheses. All atoms were refined anisotropically. After that, all hydrogens near carbon atoms were placed geometrically and refined constrained in the riding mode. For (HpipeH_2_)I(I_3_), hydrogen atoms bound to nitrogen atoms were located after an alternative series of least-squares cycles and difference Fourier maps and were refined isotropically. For (HpipeH_2_)_3_I_6_·H_2_O, two of the hydrogen atoms bound to nitrogen N6 were found from difference Fourier syntheses. The presence of hydrogen atoms on the N6 atom did not raise any questions: The N–H distances were about 1 Å and the H^…^I distances were at 2.65–2.67 Å with the N–H^…^I angles of 139–147°. Such parameters are common for the (N)H^…^I hydrogen bonds. Some of the remaining ghost peaks could also be attributed to hydrogen atoms associated with nitrogen atoms forming (N)H^…^I hydrogen bonds, but further refinement of the structure was possible only in a rigid body model. Taking into account the requirements of electroneutrality, all remaining hydrogen atoms were placed geometrically and refined constrained in a riding mode. The coordinates of the hydrogen atoms of the water molecules were calculated geometrically, then the H–O bond length was fixed as 1 Å, the thermal parameter was set as 1.2 times of that of oxygen, and the angles were refined.

A single crystal of (HpipeH_2_)_3_(H_3_O)I_7_ was selected from the synthetic mixture. The single crystal diffraction data were measured at 100 K on a Bruker D8 VENTURE with PHOTON 100 CMOS detector system equipped with a Mo-target X-ray tube (Bruker, Karlsruhe, Germany). A frame width of 0.50° and an exposure time of 15 s/frame were employed for data collection. Data reduction and integration were performed with the Bruker software package SAINT (Version 8.38A, Bruker, Karlsruhe, Germany) [58]. Data were corrected for absorption effects using the semi-empirical methods (multi-scan) as implemented in SADABS [59]. The structure was solved by the direct methods using the SHELXTL (Version 2017/1, Institute of Inorganic Chemistry, Göttingen, Germany) program package [60], which gave positions of iodine atoms. Positions of nitrogen and carbon atoms were found from successive difference Fourier syntheses. The hydrogen atoms of the HpipeH^+^ cations were calculated and further refined using riding models. The hydrogen atom positions in H_3_O^+^ were found from successive difference Fourier syntheses. Three hydrogen atomic positions were refined freely with their isotropic atomic displacement parameters restricted to 1.5 times their parent oxygen atom equivalent isotropic atomic displacement parameter. Three H–O bond distances were restrained to 0.82 Å with an estimated standard deviation of 0.02 Å. The crystal structure was refined in anisotropic approximations of atomic displacement parameters for all atoms except hydrogens.

The summary of experimental and crystallographic information for studied compounds is given Table 1. Atomic parameters are listed in Electronic Supporting Information (ESI). Further details of the crystal structures may be obtained from Cambridge Crystallographic Data Centre by quoting the CCDC numbers 1911474, 1911475, 1916483, and 1981548.

### 3.4. Thermal Analysis

Thermogravimetric analysis was performed using a NETZSCH 209 F1 Libra thermobalance (NETZSCH, Selb, Germany). Calibration performed with CaC_2_O_4_·2H_2_O showed that the accuracy of mass detection was better than 0.1%. Samples were heated in alumina crucibles under dry nitrogen flow up to 450 °C with the heating rate of 5 K·min^–1^. The NETZSCH Proteus Thermal Analysis program was used for the data processing.

### 3.5. Raman Spectroscopy

The Raman spectrum of (HpipeH_2_)I(I_3_) was recorded on a Renishaw In Via spectrometer (Renishaw, Wotton-under-Edge, Great Britain) with laser wavelength of *λ* = 514 nm (Ar, 50 mW). Sample investigation was performed in the backscattering geometry using a confocal microscope Leica DMLM (100’ lens) at room temperature in air. Focus distance was 250 mm, and the size of the laser beam was 20 μm. The CCD-camera (1024 × 368 pixels, Renishaw, Wotton-under-Edge, Great Britain) was used as a detector. The scale calibration was done using monocrystalline silica (521.5 cm^−1^) as a standard sample. WiRE 3.4 software (Renishaw, Wotton-under-Edge, Great Britain) was used for data processing.

### 3.6. Optical Spectroscopy

Optical diffuse reflectance spectra were recorded using a UV-vis spectrometer Perkin-Elmer Lambda 950 (Perkin-Elmer, Waltham, MA, USA) with an attached diffuse reflectance accessory. Measurements were performed at 298 K in the spectral range of 250–1200 nm, with a scanning rate of 2 nm/s using finely ground polycrystalline samples. The data were processed using the Kubelka–Munk theory approximation and linearized in the [(*k*/*s*)·*hυ*]^2^ − (*hυ*) coordinates with *hν* along the *x* axis and [(*k*/*s*)·*hν*]^2^ along the *y* axis, where *k* is the absorption coefficient, *s* is the scattering coefficient, and *h* is the Planck constant [61]. The (*k*/*s*) relation known as a remission function was calculated using the refraction data according to the literature as *k*/*s* = (1 − *R*)^2^/2*R*, where *R* is the absolute diffuse reflectance [62]. Extrapolation to *k* = 0 gives an approximate value of optical *E*_g_ of the material.

## 4. Conclusions

In this work, we have demonstrated the template effect of the HpipeH_2_^2+^ cation, which enables assembling different building blocks in polymer crystal structures through strong hydrogen bonds. By utilizing this effect, we were able to synthesize four new compounds, (HpipeH_2_)_2_Bi_2_I_10_·2H_2_O, (HpipeH_2_)_3_I_6_·H_2_O, (HpipeH_2_)I(I_3_), and (HpipeH_2_)_3_(H_3_O)I_7_, of which the former exhibits important properties as a potential light-harvesting material. It has considerable thermal stability up to 240 °C and a low band gap of 1.8 eV. We showed that these properties originate from strong hydrogen bonds that tie molecular moieties into the 3D crystal structure.

## Figures and Tables

**Table 1:** Structure Refinement Parameters.

Parameters	(HpipeH_2_)_2_Bi_2_I_10_·2H_2_O	(HpipeH_2_)I(I_3_)	(HpipeH_2_)_3_I_6_·H_2_O	(HpipeH_2_)_3_(H_3_O)I_7_
**Sum Formula**	**C_5_H_16_BiI_5_N_2_O**	**C_5_H_14_I_4_N_2_**	**C_15_H_44_I_6_N_6_O**	**C_15_H_45_I_7_N_6_O**
Crystal system	triclinic	orthorhombic	orthorhombic	monoclinic
Space Group	P-1 (№ 2)	Pbca (№ 61)	P2_1_2_1_2_1_ (№ 19)	P2_1_/c (№ 14)
*a*, Å	8.3972 (17)	10.5196 (3)	10.3687 (4)	24.3883 (14)
*b*, Å	10.4764 (16)	12.5020 (4)	12.1510 (3)	10.0774 (6)
*c*, Å	11.0662 (18)	21.5362 (5)	25.3425 (6)	13.6539 (8)
*α*,°	95.928 (13)	90	90	90
*β*,°	98.804 (15)	90	90	99.6360 (10)
*γ*,°	108.262 (14)	90	90	90
*V*, Å^3^	901.7 (3)	2832.36 (14)	3192.90 (16)	3308.4 (3)
*Z*	2	8	4	4
*d*_calc_	3.549	2.860	2.259	2.437
Radiation/wavelength	AgKα/0.56083	CuKα/1.54186	CuKα/1.54186	MoKα/0.71073
Temperature, K	295 (2)	293 (2)	293 (2)	100 (2)
Crystal form	block	block	plate	plate
Crystal size, mm	0.38 × 0.26 × 0.18	0.25 × 0.2 × 0.15	0.1 × 0.05 × 0.03	0.16 × 0.09 × 0.02
Absorption correction	psi-scan	multi-scan	multi-scan	multi-scan
*θ* range (data collection)	2.03–24.96	4.106–72.703	3.49–72.93	2.743–29.414
Range of *h*, *k*, *l*	–11→*h*→11; –15→*k*→15; 0→*l*→16	–12→*h*→13; –7→*k*→12; –25→*l*→26	–12→*h*→12; 0→*k*→15; 0→*l*→31	–33→*h*→33; –13→*k*→13; –18→*l*→18
*R*_int_	0.0157	0.1176	0.1467	0.0459
*R*/*R_w_*	0.0310/0.0607 ^1^	0.0399/0.1150 ^1^	0.0475/0.1203 ^1^	0.0299/0.0531 ^2^
GoF	1.09	0.992	0.899	1.079
No. of params./reflections	126/4987	116/2806	260/6313	271/9143
*Δρ*_max_ (e/Å^-3^) positive/negative	0.89/–0.72	0.93/–1.48	1.19/–0.68	1.132/–1.477

^1^ (*I* > 3*σ*(*I*)); ^2^ (*I* > 2*σ*(*I*)).

**Table 2:** Selected Interatomic Distances and Angles in the Anionic Part of the Crystal Structures of (HpipeH_2_)_2_Bi_2_I_10_·2H_2_O and (HpipeH_2_)I(I_3_).

Atoms	Distance, Å	Atoms	Angle, °
(HpipeH_2_)_2_Bi_2_I_10_·2H_2_O			
Bi1–I1 –I1 –I2 –I3 –I4 –I5	3.1459 (10) 3.1947 (17) 3.1227 (10) 3.0345 (9) 3.0342 (10) 2.9750 (16)	I1—Bi1—I1 I1—Bi1—I2 I1—Bi1—I3 I1—Bi1—I4 I1—Bi1—I5 I1—Bi1—I5 I2—Bi1—I3 I2—Bi1—I4 I2—Bi1—I5 I3—Bi1—I4 I3—Bi1—I5 I4—Bi1—I5	86.23 (2) 88.96 (2) 87.03 (2) 92.07 (2) 87.57 (2) 175.912 (18) 91.10 (2) 85.07 (2) 171.09 (2) 174.427 (19) 87.81 (2) 94.69 (2) 90.90 (2) × 2 90.86 (2) 97.70 (2)
(HpipeH_2_)I(I_3_)			
I1–I3	2.8679 (8)	I3—I1—I4	178.82 (2)
I1–I4	2.9651 (8)		

**Table 3:** Hydrogen Bonding in the Crystal Structures.

D–H···A	d(H···A), Å	d(D···A), Å	angle (D–H···A), °
(HpipeH_2_)_2_Bi_2_I_10_			
N1–H12···I2	2.79	3.543 (8)	139.5
N5–H52···I3	2.83	3.577 (6)	139.5
N1–H12···I4	2.78	3.612 (7)	150.3
N5–H51···O1	1.95	2.840 (10)	161.2
O1–H1···I5	2.79 (9)	3.725 (8)	165 (8)
(HpipeH_2_)I(I_3_)			
N2–H20···I2	2.50 (10)	3.532 (7)	165 (10)
N2–H22···I4	2.56 (19)	3.551 (7)	149 (11)
N1–H11···I2	2.80 (11)	3.570 (6)	124 (5)
N1–H12···I2	2.44 (10)	3.504 (7)	168 (8)
(HpipeH_2_)_3_I_6_·H_2_O			
N1–H1A···I3	2.637	3.520 (11)	167.3
N1–H1B···I1	2.720	3.480 (10)	142.9
N2–H2A···I2	2.783	3.533 (10)	141.7
N2–H2B···I1	2.711	3.517 (10)	149.5
N3–H3A···I6	2.863	3.606 (11)	140.9
N3–H3B···O1	1.880	2.772 (14)	170.8
N4–H4A···I1	2.832	3.577 (10)	141.0
N4–H4B···I2	2.733	3.510 (10)	145.1
N5–H5A···I4	2.739	3.547 (11)	150.0
N5–H5B···I4	2.835	3.607 (12)	144.7
N6–H6A···I5	2.653	3.539 (13)	168.3
N6–H6B···I2	2.718	3.540 (13)	152.3
O1–H1···I3	2.65 (9)	3.515 (10)	144 (12)
O1–H2···I5	2.70 (7)	3.630 (11)	155 (11)
(HpipeH_2_)_3_(H_3_O)I_7_			
N0AA–H0AA···I7	2.689	3.508 (3)	150.2
N0AA–H0AB···I7	2.744	3.586 (3)	149.1
N5–H5A···I3	2.649	3.542 (3)	167.2
N5–H5B···I2	2.822	3.542 (3)	136.9
N2–H2C···I6	2.706	3.515 (3)	148.7
N2–H2D···I6	2.718	3.539 (3)	150.5
N3–H3C···I5	2.656	3.545 (3)	165.9
N3–H3D···I2	2.831	3.521 (3)	133.6
N4–H4C···I1	2.726	3.612 (3)	165.0
N4–H4D···I4	2.870	3.532 (3)	130.8
N6–H6C···I1	2.933	3.491 (3)	121.1
N6–H6D···I4	2.866	3.677 (4)	149.2
O1–H1D···I2	2.609	3.467 (4)	170.8
O1–H1E···I3	2.796	3.605 (4)	156.7

## Supplementary Materials

The following are available online, Figure S1: X-ray powder diffraction pattern for (HpipeH_2_)_2_Bi_2_I_10_·2H_2_O, Figure S2: Thermal analysis data for (HpipeH_2_)_2_Bi_2_I_10_·2H_2_O, Figure S3: Thermal analysis data for (HpipeH_2_)_3_I_6_·H_2_O, Figure S4: Thermal analysis data for (HpipeH_2_)_3_I_6_·H_2_O, Figure S5: Thermal analysis data for (HpipeH_2_)_3_(H_3_O)I_7_ with a slight admixture of (HpipeH_2_)_3_I_6_·H_2_O Table S1: Atomic parameters in the crystal structure of (HpipeH_2_)_2_Bi_2_I_10_·2H_2_O, Table S2: Atomic parameters in the crystal structure of (HpipeH_2_)I(I_3_), Table S3: Atomic parameters in the crystal structure of ((HpipeH_2_)_3_I_6_·H_2_O, Table S4: Atomic parameters in the crystal structure of (HpipeH_2_)_3_(H_3_O)I_7_.
