# Supplementary material for: Assembling Polyiodides and Iodobismuthates Using a Template Effect of a Cyclic Diammonium Cation and Formation of a Low-Gap Hybrid Iodobismuthate with High Thermal Stability
Source: Molecules. 2020 Jun 15;25(12):2765. doi: 10.3390/molecules25122765 (PMC7355432; doi:10.3390/molecules25122765)
Supplement: Supplementary file 1 [file molecules-25-02765-s001.pdf]

molecules

MDPI

Supplementary materials

Assembling polyiodides and iodobismuthates using a template effect of a cyclic diammonium cation and formation of a low-gap hybrid iodobismuthate with high thermal stability

Tatiana A. Shestimerova,[1] Andrei V. Mironov,[1] Mikhail A. Bykov,[1] Anastasia V. Grigorieva,[1,2] Zheng Wei,[3] Evgeny V. Dikarev,[3] and Andrei V. Shevelkov[1,*]

[1] Department of Chemistry, Lomonosov Moscow State University, Moscow, 119991, Russia;
[2] Department of Materials Sciences, Lomonosov Moscow State University, Moscow, 119991, Russia;
[3] Department of Chemistry, University at Albany, Albany, NY 12222, USA;
* Correspondence: shev@inorg.chem.msu.ru;

Table S1. Atomic parameters in the crystal structure of $(HpipeH_2)_2Bi_2I_{10}\cdot 2H_2O$

Atom	*x*	*y*	*z*	U_{eq}, Å²
Bi1	0.34609(3)	0.31670(2)	0.84601(2)	0.03214(9)
I1	0.26120(6)	0.51922(4)	1.03557(4)	0.03330(14)
I2	0.37409(7)	0.52498(5)	0.66111(4)	0.04150(17)
I3	0.35660(6)	0.12452(5)	1.03197(5)	0.03860(15)
I4	0.44595(7)	0.13889(5)	0.65818(5)	0.04609(18)
I5	-0.03133(6)	0.18710(5)	0.76921(5)	0.04016(16)
N1	0.2282(9)	0.8035(7)	0.7214(7)	0.051(3)
C2	0.1066(12)	0.7631(9)	0.5973(8)	0.053(3)
C3	-0.0175(11)	0.8422(8)	0.5909(7)	0.046(3)
C4	-0.1799(10)	0.7813(8)	0.6364(7)	0.042(2)
N5	-0.1592(7)	0.7250(6)	0.7562(5)	0.0338(17)
C6	-0.0011(9)	0.8034(7)	0.8468(6)	0.039(2)
C7	0.1490(9)	0.7580(7)	0.8285(7)	0.042(2)
H21	0.043792	0.667343	0.584527	0.0631
H22	0.170662	0.780528	0.532411	0.0631
H31	-0.044563	0.858326	0.507648	0.0546
H32	0.040215	0.933395	0.634153	0.0546
H41	-0.259877	0.711262	0.573104	0.0501
H42	-0.236209	0.847628	0.645243	0.0501
H61	-0.020973	0.795299	0.929213	0.0465
H62	0.027073	0.898506	0.841077	0.0465
H71	0.114717	0.660598	0.820237	0.0505
H72	0.234757	0.788015	0.903067	0.0505
H11	0.317624	0.771364	0.716903	0.0612
H12	0.280774	0.896551	0.736986	0.0612

H51	-0.163772	0.635968	0.738974	0.0405
H52	-0.252538	0.719327	0.79206	0.0405
O1	0.1663(9)	0.5356(7)	0.3505(6)	0.0591(15)
H1	0.141(11)	0.603(8)	0.306(9)	0.0706
H2	0.289(4)	0.565(12)	0.369(10)	0.0706

Table S2. Atomic parameters in the crystal structure of (HpipeH$_2$)I(I$_3$)

Atom	*x*	*y*	*z*	U_{eq}, Å^2
I1	0.40775(5)	0.62933(4)	0.58872(2)	0.06347(17)
I2	0.69860(5)	0.47157(4)	0.73975(2)	0.06655(17)
I3	0.58854(6)	0.79043(4)	0.62320(3)	0.07574(19)
I4	0.22288(6)	0.45991(5)	0.55514(3)	0.0790(2)
N1	0.5409(7)	0.0890(5)	0.6646(3)	0.0604(14)
N2	0.4591(8)	0.3440(6)	0.6519(3)	0.0655(15)
C1	0.5825(9)	0.1150(7)	0.5994(3)	0.0672(19)
H1A	0.5743	0.0513	0.5740	0.081
H1B	0.6717	0.1348	0.6001	0.081
C2	0.5356(9)	0.1793(7)	0.7082(3)	0.0632(17)
H2A	0.6149	0.2186	0.7059	0.076
H2B	0.5275	0.1517	0.7501	0.076
C3	0.4263(9)	0.2557(7)	0.6956(4)	0.0684(18)
H3A	0.3556	0.2155	0.6785	0.082
H3B	0.3985	0.2865	0.7346	0.082
C4	0.5333(9)	0.3155(7)	0.5945(4)	0.0700(19)
H4A	0.6232	0.3213	0.6038	0.084
H4B	0.5139	0.3672	0.5623	0.084
C5	0.5072(10)	0.2049(6)	0.5696(3)	0.070(2)
H5A	0.5249	0.2048	0.5254	0.084
H5B	0.4174	0.1897	0.5748	0.084
H20	0.526(9)	0.394(7)	0.674(4)	0.07(2)
H21	0.36(2)	0.360(12)	0.633(9)	0.17(6)
H11	0.447(10)	0.047(7)	0.660(4)	0.07(3)
H12	0.617(12)	0.043(9)	0.684(6)	0.10(4)

Table S3. Atomic parameters in the crystal structure of (HpipeH$_2$)$_3$I$_6$·H$_2$O

Atom	*x*	*y*	*z*	U_{eq}, Å^2
I1	0.50187(10)	0.92179(7)	0.15106(3)	0.0814(2)
I2	0.44274(9)	0.92538(7)	0.48195(4)	0.0810(2)
I3	0.84255(9)	0.73158(7)	0.69052(3)	0.0829(2)
I4	0.51422(11)	0.43196(8)	0.68170(3)	0.0903(3)
I5	0.30075(10)	0.77848(9)	0.66295(4)	0.0938(3)

I6	0.68140(11)	0.23954(8)	0.52755(4)	0.1031(3)
N1	0.4652(11)	1.0301(9)	0.2773(4)	0.080(3)
H1A	0.4211	0.9688	0.2858	0.096
H1B	0.4630	1.0362	0.2419	0.096
N2	0.4751(11)	1.1484(8)	0.3934(4)	0.075(3)
H2A	0.4576	1.1218	0.4258	0.090
H2B	0.4711	1.2222	0.3955	0.090
N3	0.5373(10)	0.5086(9)	0.5388(4)	0.075(3)
H3A	0.5679	0.4470	0.5541	0.091
H3B	0.5650	0.5662	0.5580	0.091
N4	0.4653(12)	0.6552(8)	0.4339(4)	0.082(3)
H4A	0.4755	0.6268	0.4014	0.098
H4B	0.4571	0.7285	0.4301	0.098
N5	0.5076(13)	0.6544(9)	0.7759(5)	0.086(3)
H5A	0.5039	0.7281	0.7728	0.103
H5B	0.5174	0.6268	0.7432	0.103
N6	0.5568(14)	0.5224(11)	0.8863(6)	0.104(4)
H6A	0.6035	0.4669	0.8727	0.124
H6B	0.5696	0.5221	0.9215	0.124
O1	0.5958(10)	0.6950(8)	0.5971(4)	0.089(3)
H1	0.632(14)	0.694(12)	0.634(2)	0.107
H2	0.513(8)	0.734(11)	0.605(6)	0.107
C1	0.3950(16)	1.1266(11)	0.3001(4)	0.082(4)
H1C	0.4461	1.1927	0.2952	0.099
H1D	0.3138	1.1364	0.2817	0.099
C2	0.3700(13)	1.1104(11)	0.3567(5)	0.074(3)
H2C	0.3551	1.0327	0.3628	0.088
H2D	0.2912	1.1491	0.3658	0.088
C3	0.5993(15)	1.0140(13)	0.2930(6)	0.089(4)
H3C	0.6501	1.0732	0.2779	0.107
H3D	0.6295	0.9458	0.2774	0.107
C4	0.6260(15)	1.0099(12)	0.3509(6)	0.090(4)
H4C	0.5675	0.9572	0.3669	0.108
H4D	0.7130	0.9826	0.3561	0.108
C5	0.6132(16)	1.1167(11)	0.3796(6)	0.089(4)

H5C	0.6503	1.1746	0.3581	0.107
H5D	0.6630	1.1127	0.4120	0.107
C6	0.5932(14)	0.5172(12)	0.4848(6)	0.088(4)
H6C	0.5470	0.4684	0.4612	0.105
H6D	0.6827	0.4940	0.4857	0.105
C7	0.5853(14)	0.6339(10)	0.4640(5)	0.080(3)
H7A	0.5899	0.6848	0.4935	0.096
H7B	0.6590	0.6478	0.4414	0.096
C8	0.3949(13)	0.5058(12)	0.5409(6)	0.088(4)
H8A	0.3674	0.5015	0.5774	0.105
H8B	0.3642	0.4403	0.5230	0.105
C9	0.3334(14)	0.6094(14)	0.5148(7)	0.097(4)
H9A	0.2435	0.6135	0.5252	0.117
H9B	0.3763	0.6744	0.5284	0.117
C10	0.3406(14)	0.6118(12)	0.4558(6)	0.088(4)
H10A	0.2705	0.6569	0.4426	0.106
H10B	0.3276	0.5376	0.4427	0.106
C11	0.6253(14)	0.6269(16)	0.8066(5)	0.097(5)
H11A	0.6926	0.6789	0.7973	0.116
H11B	0.6545	0.5543	0.7961	0.116
C12	0.6084(19)	0.6281(15)	0.8649(7)	0.111(5)
H12A	0.5499	0.6872	0.8742	0.133
H12B	0.6910	0.6433	0.8813	0.133
C13	0.3814(17)	0.6162(12)	0.7964(7)	0.101(5)
H13A	0.3195	0.6164	0.7677	0.121
H13B	0.3515	0.6680	0.8228	0.121
C14	0.3844(17)	0.5042(13)	0.8202(6)	0.093(4)
H14A	0.2996	0.4715	0.8164	0.112
H14B	0.4445	0.4594	0.8003	0.112
C15	0.4211(17)	0.4996(13)	0.8765(6)	0.096(5)
H15A	0.3694	0.5524	0.8959	0.115
H15B	0.4009	0.4269	0.8900	0.115

Table S4. Atomic parameters in the crystal structure of $(HpipeH_2)_3(H_3O)I_7$

Atom	*x*	*y*	*z*	U_{eq}, Å^2
I1	0.16128(2)	0.46074(2)	0.42215(2)	0.01058(5)
I2	0.24807(2)	-0.21084(3)	0.25672(2)	0.01181(5)
I3	0.12683(2)	-0.05899(3)	0.44480(2)	0.01370(6)
I4	0.34244(2)	0.03854(2)	0.07972(2)	0.01140(5)
I5	0.36631(2)	0.54326(3)	0.05030(2)	0.01481(6)
I6	0.43530(2)	0.25844(2)	0.35414(2)	0.01143(5)
I7	0.06797(2)	0.24559(2)	0.14580(2)	0.01169(6)
O1	0.25011(15)	0.7603(3)	0.0043(3)	0.0267(7)
N0AA	0.02945(13)	0.2236(3)	0.3816(2)	0.0105(6)
H0AA	0.052002	0.218604	0.334913	0.013
H0AB	0.051281	0.213491	0.442025	0.013
N2	0.47106(13)	0.2700(3)	0.1154(2)	0.0099(6)
H2C	0.447958	0.271116	0.161240	0.012
H2D	0.449416	0.274926	0.054337	0.012
N3	0.60691(13)	0.3282(3)	0.1788(2)	0.0113(6)
H3C	0.612388	0.375905	0.124640	0.014
H3D	0.636789	0.342451	0.227229	0.014
N4	0.28449(14)	0.3201(3)	0.3488(2)	0.0128(7)
H4C	0.256938	0.352205	0.379623	0.015
H4D	0.316870	0.326089	0.392683	0.015
N5	-0.10795(13)	0.1942(3)	0.3237(2)	0.0123(7)
H5A	-0.114043	0.146671	0.377546	0.015
H5B	-0.138906	0.186341	0.276558	0.015
N6	0.21476(15)	0.1641(3)	0.1572(2)	0.0171(7)
H6C	0.180115	0.140688	0.126374	0.020
H6D	0.238717	0.101840	0.140913	0.020
C1	0.50096(16)	0.1397(4)	0.1230(3)	0.0119(8)
H1A	0.475912	0.071234	0.088047	0.014
H1B	0.509846	0.113866	0.193872	0.014
C2	-0.01062(16)	0.1099(4)	0.3658(3)	0.0121(8)
H2A	-0.024180	0.091956	0.428916	0.015
H2B	0.009516	0.029880	0.349156	0.015
C3	-0.10161(15)	0.3371(4)	0.3534(3)	0.0119(7)

H3A	-0.133573	0.363243	0.385287	0.014
H3B	-0.102862	0.391770	0.292813	0.014
C4	0.50686(16)	0.3915(4)	0.1299(3)	0.0124(8)
H4A	0.483571	0.467805	0.143102	0.015
H4B	0.520957	0.410161	0.067413	0.015
C5	0.60560(16)	0.1846(4)	0.1520(3)	0.0141(8)
H5C	0.607501	0.131599	0.213466	0.017
H5D	0.639118	0.163778	0.122786	0.017
C6	0.00427(15)	0.3604(4)	0.3771(3)	0.0113(7)
H6A	-0.004902	0.388372	0.306724	0.014
H6B	0.032117	0.423388	0.411624	0.014
C7	0.22922(18)	0.2951(4)	0.1130(3)	0.0166(8)
H7A	0.264046	0.283452	0.085726	0.020
H7B	0.199406	0.317647	0.056965	0.020
C8	-0.04821(15)	0.3678(4)	0.4241(3)	0.0120(7)
H8A	-0.044483	0.304534	0.480293	0.014
H8B	-0.051057	0.458027	0.451403	0.014
C9	0.55615(15)	0.3815(4)	0.2141(3)	0.0111(7)
H9A	0.564511	0.470445	0.243572	0.013
H9B	0.546332	0.322499	0.266491	0.013
C10	-0.06051(16)	0.1321(4)	0.2845(3)	0.0136(8)
H10A	-0.049477	0.190187	0.232610	0.016
H10B	-0.072604	0.045972	0.253273	0.016
C11	0.27304(17)	0.1771(4)	0.3262(3)	0.0150(8)
H11A	0.276552	0.126932	0.389259	0.018
H11B	0.301201	0.142369	0.288341	0.018
C12	0.55464(15)	0.1423(4)	0.0794(3)	0.0124(8)
H12A	0.561268	0.052587	0.054295	0.015
H12B	0.549663	0.203745	0.022026	0.015
C13	0.23662(16)	0.4099(4)	0.1847(3)	0.0129(8)
H13A	0.204055	0.412824	0.219267	0.015
H13B	0.236535	0.493015	0.146004	0.015
C14	0.21546(17)	0.1547(4)	0.2666(3)	0.0143(8)
H14A	0.202040	0.065817	0.282461	0.017
H14B	0.189598	0.221487	0.286290	0.017

C15	0.28888(17)	0.4074(4)	0.2625(3)	0.0188(9)
H15A	0.320359	0.376778	0.230973	0.023
H15B	0.297315	0.498934	0.286819	0.023
H1C	0.250(2)	0.830(3)	-0.034(3)	0.028
H1D	0.252(2)	0.776(5)	0.0673(16)	0.028
H1E	0.2242(16)	0.711(4)	-0.027(3)	0.028

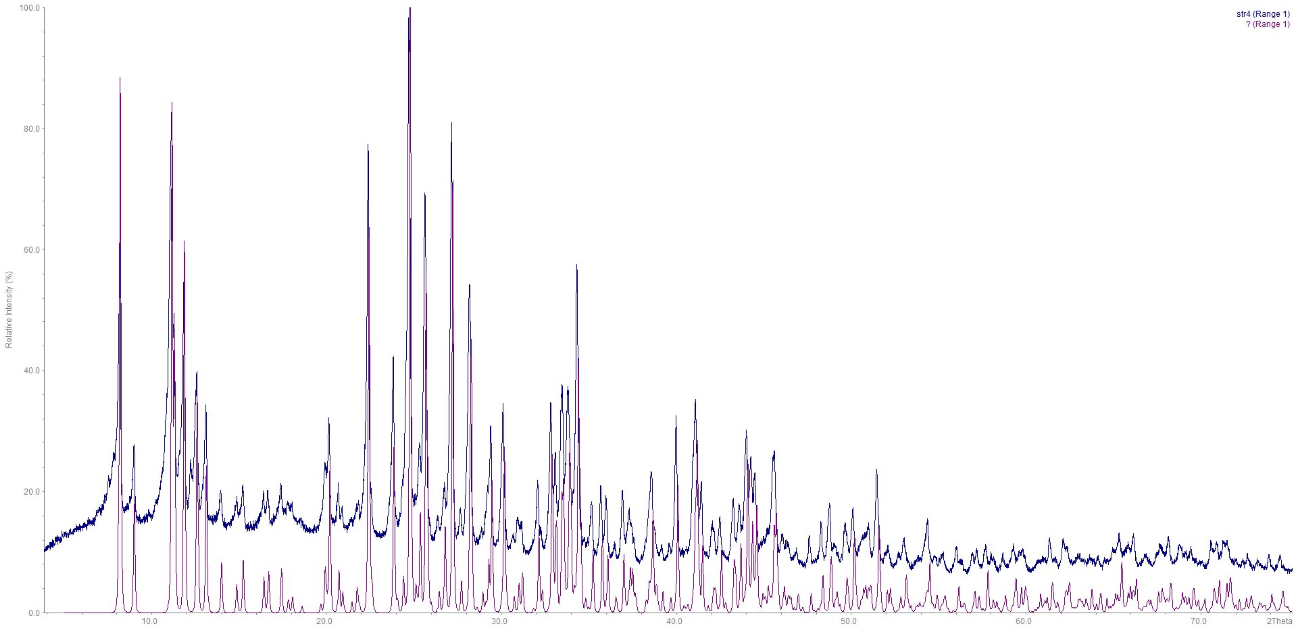

Figure 1. X-ray powder diffraction pattern for $(HpipeH_2)_2Bi_2I_{10}\cdot 2H_2O$. Experimental profile, blue; calculated from the crystal data, magenta.

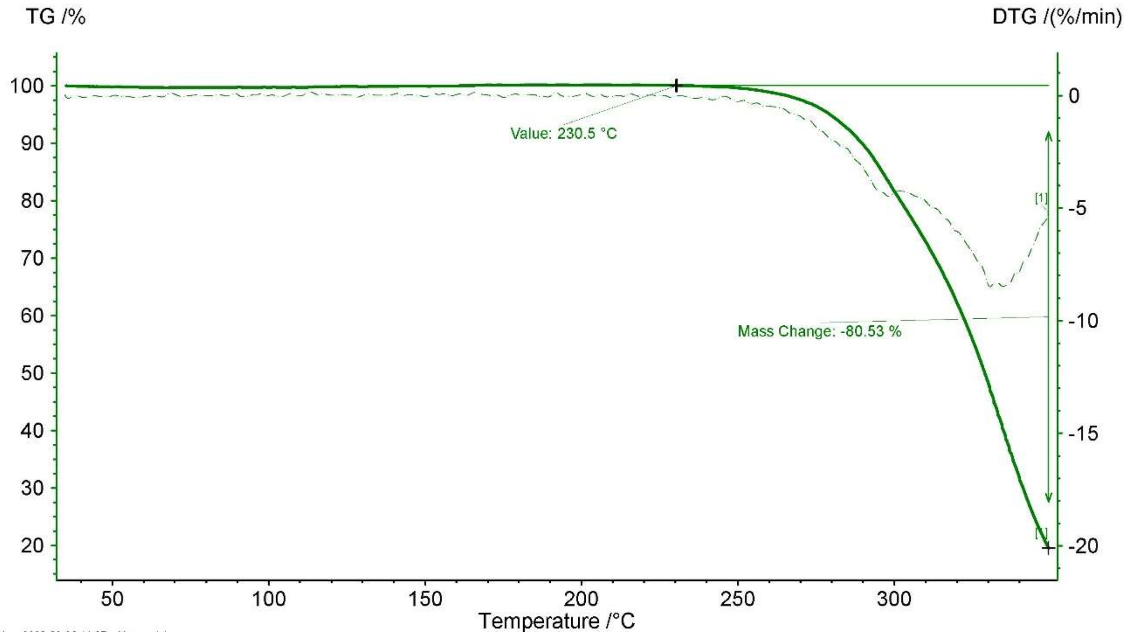

Figure 2. Thermal analysis data for $(HpipeH_2)_2Bi_2I_{10}\cdot 2H_2O$.

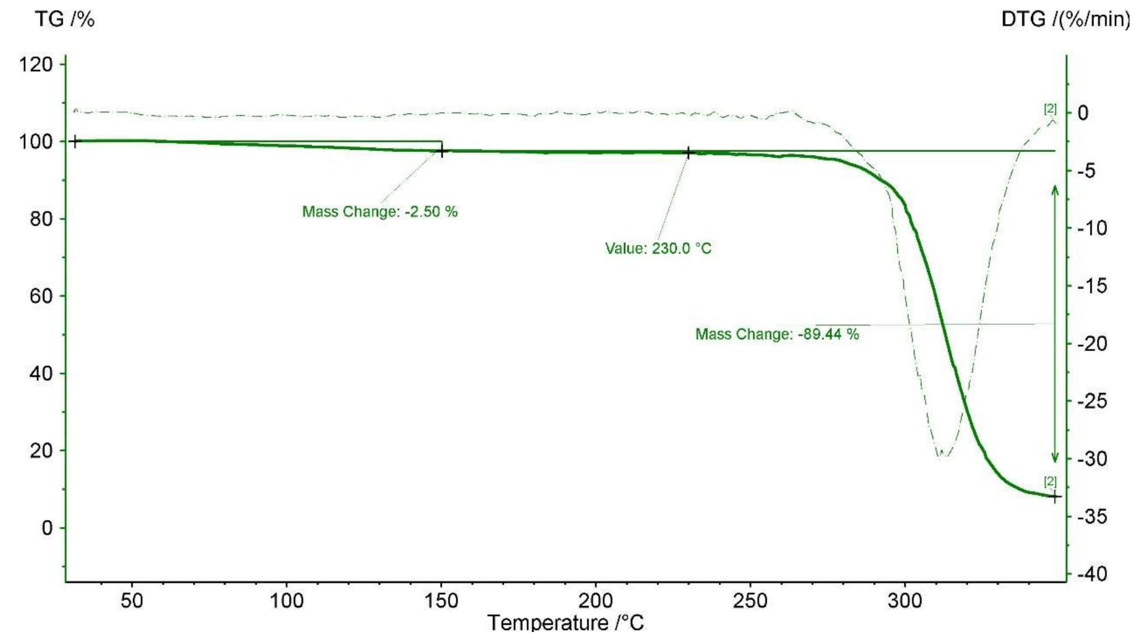

Figure 3. Thermal analysis data for $(HpipeH_2)_3I_6{\cdot}H_2O$.

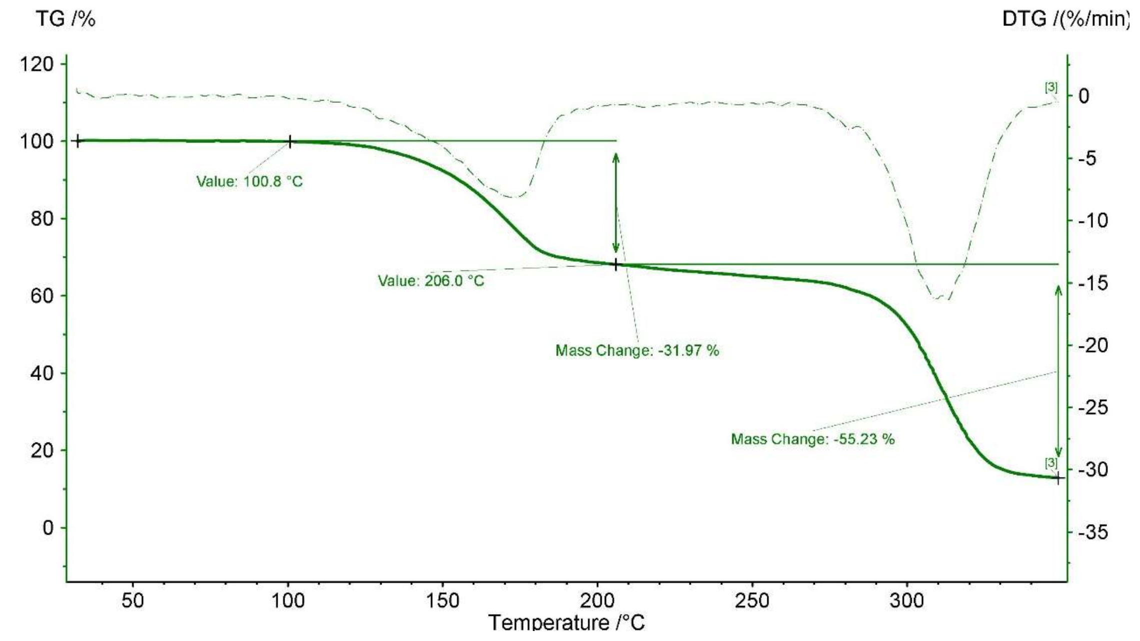

Figure 4. Thermal analysis data for $(HpipeH_2)I(I_3)$.

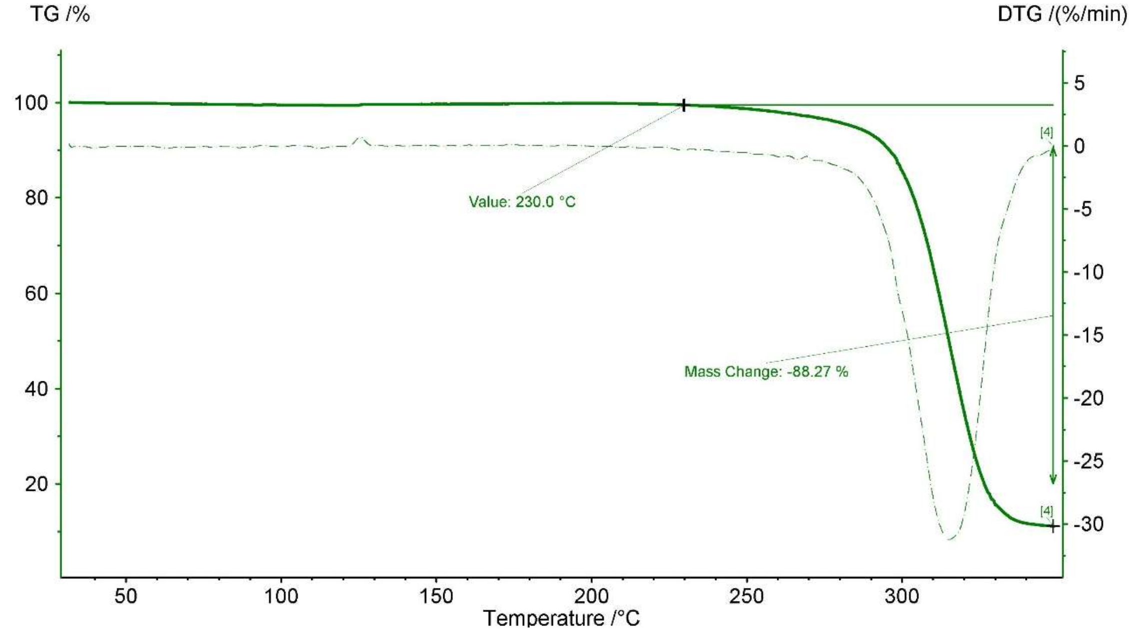

Figure 5. Thermal analysis data for $(HpipeH_2)_3(H_3O)I_7$ with a slight admixture of $(HpipeH_2)_3I_6\cdot H_2O$.
